# Notes and News

**Published:** 1889-04-06

**Authors:** 


### NOTES AND NEWS.

COLLECTED AND COMMUNICATED.

The Marquis of Carmarthen, M.P., has been appointed chairman of the council of the Hospitals Association, vice Major Alexander Ross, M.P., deceased.

The greatest hospital function of the century will be performed in May next, when the Johns Hopkins Hospital will be opened at Baltimore.

The annual meeting of Cheltenham Hospital Governors was held on the 19th ult. The expenditure last year was £4,777, including £300 spent on new work. The income was £4,727. The average cost of each in-patient is estimated at £4 1s. 2d., and of each out-patient at 3s. 4½d. This is lower than last year, and shows careful management. An epidemic of typhoid which occurred at Cheltenham last year severely taxed the resources of the hospital, but Miss Tatham, the matron, proved a skilful director, and rose to the occasion. Her efforts are appreciated and recognised by the committee.

It is reported that the use of opiates by Parisian ladies is rapidly increasing, and the attention of the Academy of Medicine has been called to this subject. The injection of morphia was the latest form in which a remedy for *ennui* was sought, but a new drug called "exalgine" has been introduced, which is even more deleterious than morphia. Even in England the morphia habit is increasing in fashionable circles, and working much misery in many families. Physicians and nurses cannot be too careful not to instruct the laity in the use of the hypodermic syringe unless it is absolutely necessary.

The annual meeting of Bristol Royal Infirmary supporters was held last week. The committee had to report a diminution of 2,288 in the number of out-patients, as they have found it necessary to enforce more strictly the rule as to obtaining a note of admission. The number of in-patients was more last year than in the preceding year by 25, and the cost per patient was less, being £3 0s. 3d. as against £3 5s. in 1887. The death rate was the lowest on record, being only 4·41 per cent., as against 5·6 in 1887. The funds are rather better than last year, but still the fixed income does not nearly meet the expenses, and more subscribers are badly needed.

We are always glad to receive reports of The Firs Home, Bournemouth, though we are sorry to see that its funds at the present time are at a low ebb. The home is for patients in advanced consumption, and a distinctive feature of the management is that no interest is required to get a patient in. They are admitted on the medical merits of the case, and the only requirement is that the weekly payment of 10s. 6d. must be guaranteed. There are twenty beds in the home, ten for men and ten for women. The lady-superintendent, Miss Poole, sees that the patients have every possible attention and comfort, and an excellent medical staff afford such alleviation of suffering as is possible.

The story of the madness of William Rob, the composer, seems to be that prosperity is not always a blessing. In his younger days Rob was a thorough bohemian, and knew the poverty and pleasures, the bodily hunger and intellectual fulness of that queer phase of life. But Baron de Rothschild heard Rob play, and, struck by his genius, carried him off to be his private chief musician. Rob was introduced to the Prince of Wales, travelled far and wide, knew want no longer, but his brain began to give way. He developed a liking for bathing in champagne and generally misusing the luxuries which now surrounded him. Ultimately he had to be locked up in an asylum. Prosperity had driven him mad.

The annual meeting of friends of University College Hospital took place on March 27, Mr. Augustus Prevost presiding. The annual report was received and adopted. It called urgent attention to the question of rebuilding, which the medical staff considered would soon become a matter of necessity. Contributions had been received or promised to the amount of £3,234, but it had been decided not to commence rebuilding until £30,000 had been received or promised. The state of the finances caused the committee much anxiety. The small reserve of investments had been trenched upon, and at the end of the year the debt was no less than £18,419 4s. 10d. Only six beds have yet been used at the Wakley Convalescent Home, as the committee need to proceed tentatively owing to their financial straits.

Some experiments have lately been carried on at Hyderabad Medical School to prove whether chloroform, in any circumstances, directly affects the heart or not. The experiments were made on 128 pariah dogs, and the conclusion arrived at was that, no matter in what way it was given, in no case did the heart become dangerously affected by chloroform until after the breathing had stopped. The Principal of the School, Dr. Lawrie, says that chloroform is there administered by students with absolute safety; and they are never allowed to examine the heart beforehand, nor to feel the pulse during the administration. This is a theory worthy of consideration, for if warnings are always given by the breathing, the danger of an overdose of chloroform can always be avoided by attention to the respiration of the patient. So long as the present law holds in England though, the administrator of chloroform must be ready to swear, in case of accident, that he examined both pulse and heart, or he will be held responsible for any evil effects.

The following vacancies are announced this week, the amount of the salary in each case being given after the name of the institution:

**Resident Medical Officers.**

Bristol General Hospital. House Surgeon.
City of London Hospital, Victoria Park. House Physician.
Fisherton Asylum, Salisbury. Assistant Medical Officer.—£100, board, &c.
Hospital for Consumption, Brompton. House Physician.
Metropolitan Hospital. Senior House Surgeon. — £80, board, &c.
Metropolitan Hospital. Junior House Surgeon. — £40, board, &c.
Monkwearmouth Dispensary. House Surgeon. — £80, board, &c.
Hospital for Women, Soho-square. Two clinical assistants.
Scarborough Friendly Society. Medical Officer.—£200, residence, &c.

**Non-Resident Medical Officers.**

Middlesex Hospital. Obstetric Physician.
Salford Royal Hospital. Assistant Surgeon.
Enniskillen Union. Medical Officer.—£80.
Parish of Arrochar. Medical Officer.—£100.

The Bread and Food Reform League has, as one of its chief aims, the supplying of good nutritious food, at the lowest reasonable cost, to the poor of the metropolis and elsewhere. Unluckily, in London there are generally half-a-dozen different societies with similar ideas, but contrary methods, all fighting in the same field. Here are the Poor Children's Aid Society, the Penny Dinner Council, the Sanitary Institute, and numerous public and private charities, all interfering with the trade principles of the Bread and Food Reform League. Very naturally the League wishes to co-operate with these other societies, and if this is done the power of the League, and its usefulness, will be greatly increased.

Dr. Richardson seems very well satisfied with the pulse-reading and cough-reading powers of the graphophone with which he has been experimenting. We have already looked forward to the delightful time when, however far from our pet doctor, we shall be able to send him a specimen of our cough, etc., by post, and receive in return a diagnosis of our case. But Dr. Richardson has got another idea; he thinks, for teaching purposes the graphophone will be invaluable, as the poor patient will not be obliged to submit to being examined by some dozens of students, all of whom will turn their attention to the graphophone, from whence the record, once written, can be repeatedly indefinitely without even the presence of the patient.

A meeting of medical men was held at Birmingham last week to consider how the abuse of the "Queen's," "General," and "Eye" Hospitals was to be overcome and prevented. Mr. Cheshire presided; Mr. Marsh acted as secretary. It is one of the difficulties of the penny-a-week plan that respectable artisans who subscribe to it think they have, therefore, a right to get all their ailments and those of their families attended to at the different hospitals. Why shouldn't every subscriber have the right to be a patient? Well, when the subscriber earns from two to four pounds a week and only "subscribes" to the penny plan, we think the right is questionable. What with friendly societies, provident dispensaries, abuse of hospitals, and the ever-increasing rush of students to the medical schools, the life of the general practitioner is not free from apprehension of future starvation. Still, is it not a little *infra. dig.* to make a fuss about probable starvation? It is not only the medical profession that is overcrowded, it is not only hospitals which are subject to abuse. If the score of physicians and surgeons who discussed the subject at Birmingham had arrived at any useful conclusion on how to remedy present evils it would be a different matter; but they only decided to form a committee and ask the advice of chairmen of different hospitals.

A scheme has been formulated for the supply of salt water to the Royal Sea Bathing Infirmary at Scarborough. As this will necessitate the laying of pipes across the sands, the Town Council have had to be consulted on the matter. It is to be hoped they will give not only consent, but monetary support to the scheme, for at present the main contributions are received fron non-residents; for whilst the total amount of subscriptions last year amounted to £685, only £74 was received from residents in Scarborough. The benefit obtained from a residence at the seaside by the 563 poor patients who were admitted last year, and who without such provision would have been debarred the privilege, is incalculable. The institution is a credit and honour to the town, and its inhabitants should further its interests in every way possible.

The Mayor of Barnsley laid the foundation-stone of the Kendray Fever Hospital on March 26. The hospital is being built at a cost of £5,000 by Mrs. A. A. Lambert, in memory of her father, the late Francis Kendray, of Barnsley. The hospital consists of an administrative department, an isolated block, a ward for smallpox patients, and the usual wash-house and laundry buildings. The administrative block consists of two wings, between which is a covered entrance archway. The isolation block provides two wards of three beds each, and two of two beds each, with nurses' room adjoining, and gives accommodation for five males and five females. An open verandah runs along the front of this block, and all proper bath and other arrangements are provided. Special arrangements will be made in the ward of this building for the complete destruction of the germs of disease by passing the foul air through heat, so as to render the vicinity of the hospital harmless, and to prevent the spread of infections to the other parts of the hospital or beyond. The arrangement of this part of the scheme is carried out according to the suggestions of the Royal Commission on Smallpox. The buildings are of a simple character—the administrative block being in the Queen Anne style. Brick, with stone dressing, is to be used, and the walls are to be plastered with Parian cement inside, and all the angles rounded. The architects are Messrs. Morley and Woodhouse, of Bradford and Bolton.

At the annual meeting of the Scarborough Hospital Governors on March 26, a letter was read from Lieutenant-Colonel Steble, offering to give £1,000 towards the purchase of a site for new buildings, if before a period of twelve months another £4,000 is promised The present building was only intended for a dispensary, and has become a hospital because of the absolute necessity for such an institution. During last year 353 patients were admitted to the hospital. Of these, 250 were cases of minor accidents, 103 required treatment in the wards, and remained during an aggregate of 2,075 days, giving an average of twenty days' stay in hospital per patient. The admissions during the year in the dispensary department were 1,993; visits by house surgeons 13,050, being an average of thirty-five daily. The work of the dispensary has been exceedingly heavy, the out-patients being so numerous as again to necessitate the appointment of an assistant house surgeon during the winter months. The number of patients admitted during the year has been 1,993. As showing the amount of work which has devolved upon the medical staff, it may be mentioned that 13,050 visits have been paid to patients at their own homes, being 3,061 in excess of the number in 1887. The hospital patients have been fewer than in the preceding year, the numbers being 353 as against 403. The treasurer's balance-sheet shows that the deficiency of £107 17s. 11d. at the commencement of the year has been increased to £188 11s. 5d., despite the very generous gift of fifty guineas from the president, Lieutenant-Colonel Steble. Surely the example of Lieutenant-Colonel Steble will find some followers in a town like Scarborough?

